# Superiority of capsaicin 8% patch versus oral pregabalin on dynamic mechanical allodynia in patients with peripheral neuropathic pain

**DOI:** 10.1002/ejp.1155

**Published:** 2017-12-01

**Authors:** G. Cruccu, T.J. Nurmikko, E. Ernault, F.K. Riaz, W.T. McBride, M. Haanpää

**Affiliations:** ^1^ Department of Neurology and Psychiatry Sapienza University Rome Italy; ^2^ The Walton Centre NHS Foundation Trust Liverpool UK; ^3^ Astellas Pharma Inc. Leiden The Netherlands; ^4^ Astellas Pharma Inc. Chertsey UK; ^5^ Belfast Health and Social Care Trust Belfast Northern Ireland; ^6^ Helsinki University Central Hospital Helsinki Finland

## Abstract

**Background:**

Dynamic Mechanical Allodynia (DMA) is a typical symptom of neuropathic pain (NP). In a recent study, the capsaicin 8% patch was noninferior to pregabalin in overall peripheral NP relief. In this study, we report the comparison of the two treatments in relieving DMA.

**Methods:**

In a randomized, open‐label, head‐to‐head, 8‐week study, 488 patients with peripheral NP were treated with the capsaicin 8% patch (one application) or an optimized dose of pregabalin. Assessments included the area and intensity of DMA, and the number of patients achieving complete resolution of DMA.

**Results:**

At baseline, 253 patients in the capsaicin 8% patch group and 235 patients in the pregabalin group had DMA. From baseline to end of study, the change in DMA intensity was significantly in favour of the capsaicin 8% patch versus pregabalin [−0.63 (95% CI: −1.04, −0.23; *p = *0.002)]. Similarly, the capsaicin 8% patch was superior to pregabalin in reducing the area of DMA [−39.5 cm^2^ (95% CI: −69.1, −10.0; *p = *0.009)] from baseline to end of study. Overall, a greater proportion of patients had a complete resolution of allodynia with capsaicin 8% patch treatment compared with pregabalin treatment (24.1% vs. 12.3%; *p = *0.001) at end of study.

**Conclusion:**

Capsaicin 8% treatment was superior to pregabalin in reducing the intensity and area of DMA, and in the number of patients with complete resolution of DMA.

**Significance:**

The superiority of a topical treatment over pregabalin in relieving DMA supports the view that both peripheral and central sensitization can mediate allodynia.

## 1 Introduction

Allodynia, a common clinical manifestation of peripheral neuropathic pain (NP), arises due to a stimulus that does not normally provoke pain and involves a change in the quality of sensation (IASP, 2014; Jensen and Finnerup, 2014). The incidence of allodynia in NP disorders varies, with prevalence estimates of 31%, 75% and 79% in painful polyneuropathy, postherpetic neuralgia (PHN) and postinjury NP, respectively (Maier et al., 2010). Dynamic mechanical allodynia (DMA) is pain evoked by light brushing or stroking of the skin (Truini et al., 2013b; Jensen and Finnerup, 2014) and may be generated by several different mechanisms.

Compelling evidence supports the widely held view that DMA represents a form of secondary hyperalgesia due to altered processing of low‐threshold large diameter A‐beta fibres in the central nervous system (CNS), commonly referred to as central (or *indirect*) sensitization (Hansson, 2014; Jensen and Finnerup, 2014). Microneurographic studies have shown that blocking of A‐receptors by compression can also block DMA both in animal and in human models (Campbell et al., 1988; Torebjork et al., 1992; Ochoa and Yarnitsky, 1993). The temporal dynamics of DMA behaviour in animal models of nerve ligature are compatible with conduction in myelinated fibres, in the absence of C‐activation (Liu et al., 2000; Sun et al., 2005). Previously proposed mechanisms of DMA, such as aberrant reinnervation by collaterals of A‐beta primary afferents on deafferented second‐order nociceptive neurons (Koltzenburg et al., 1992, 1994; Ochoa and Yarnitsky, 1993; Fields et al., 1998; Landerholm and Hansson, 2011), are nowadays thought to occur less frequently (Cervero and Laird, 1996). It is generally accepted that central sensitization is maintained in part by continuous input from nociceptors (Woolf, 2009; Baron et al., 2013). Others, who favour a peripheral (or *direct*) mechanism, believe that low‐threshold, mechano‐sensitive C fibres (C tactile fibres), may independently contribute to mechanical allodynia. Moreover, microneurography (Ochoa et al., 2005; Serra et al., 2011), laser‐evoked potential (Truini et al., 2013a), skin biopsy (Truini et al., 2014) and pharmacological studies (Haroutounian et al., 2014), consistently provide evidence suggesting that peripheral sensitization of mechanothermal nociceptors, innervated by A‐delta and C fibres (mechano‐heat units), induces lowering of the mechanical threshold to a level that allows direct excitation by innocuous stimuli, thus offering an alternative target for topical capsaicin.

We postulated that application of high‐concentration topical capsaicin, acting on C fibres that supply the pain area, would lead to a reduction in peripheral and central sensitization and subsequent attenuation of DMA. Capsaicin is a potent, highly selective vanilloid receptor subtype 1 (TRPV1) agonist that causes depolarization of the neurons, inducing a short‐lived warming sensation, followed by complete defunctionalization (Anand and Bley, 2011). The efficacy and tolerability of the capsaicin 8% patch has been confirmed in PHN, painful human immunodeficiency virus (HIV)‐associated neuropathies (Backonja et al., 2008; Simpson et al., 2008; Irving et al., 2011; Brown et al., 2013), and more recently in painful diabetic peripheral neuropathy (Simpson et al., 2016; Vinik et al., 2016).

Clinical trials specifically investigating the treatment of evoked pain are sparse and currently there are no defined standard of care treatments for DMA (Jensen and Finnerup, 2014). Pregabalin is a well‐established and widely used treatment for NP (Attal et al., 2010), which has been shown to reduce allodynia in experimental studies (Tuchman et al., 2010), and was significantly better than placebo in alleviating DMA in patients with PHN (Stacey et al., 2008). Whilst these studies have demonstrated the clinical benefit of pregabalin in allodynia, including a correlation between changes in the intensity of DMA and overall pain (Stacey et al., 2008), the relationship between changes in the area of allodynia and a clinical response remains to be explored.

In the recently completed ELEVATE study, the capsaicin 8% patch demonstrated noninferior pain relief versus an optimized dose of pregabalin over 8 weeks (primary endpoint), with a faster onset of action, fewer systemic side effects and greater patient satisfaction with treatment (Haanpää et al., 2016). Here, we report results regarding the intensity and area of DMA, which formed secondary outcome measures, in the same study population.

## 2 Methods

### 2.1 Study design and participants

The ELEVATE (NCT01713426) study was a Phase IV, randomized, open‐label, head‐to‐head, 8‐week, noninferiority study, conducted in Europe between July 2012 and September 2013 (Haanpää et al., 2016). Eligible patients were: aged 18–80 years; had a documented diagnosis of probable or definite peripheral NP due to PHN (Treede et al., 2008), peripheral nerve injury (PNI) or nondiabetic painful polyneuropathy; had an average Numeric Pain Rating Scale (NPRS) score ≥4 at screening (over at least four consecutive days); were naïve to treatment with the capsaicin 8% patch and were naïve to, or had not received adequate treatment with, pregabalin and gabapentin; and provided written consent. Exclusion criteria included the following: significant ongoing or recurrent pain of an aetiology other than PHN, PNI or nondiabetic painful polyneuropathy; Complex Regional Pain Syndrome; NP related to previously administered radiotherapy, diabetes mellitus or HIV‐associated neuropathy; or NP areas located only on the face, above the hairline of the scalp, and/or in proximity to mucous membranes. Full inclusion and exclusion criteria and patient characteristics and demographics are reported elsewhere (Haanpää et al., 2016).

### 2.2 Treatment and assessments

Patients were randomized to receive the capsaicin 8% patch (a single application, 640 μg/cm^2^, of up to four patches per application) or an optimized dose of pregabalin (150–600 mg/day administered in two or three doses) (Haanpää et al., 2016). The endpoints for DMA analysis were the change in the intensity and the area of allodynia from baseline to Week 8. Allodynia was analyzed in all randomized patients who initiated study treatment. The analyses of changes in allodynia were based on patients with allodynia at baseline, which was defined as patients with pain intensity >0 (NPRS score) and a sensitive area of allodynia >0 cm^2^.

The area of DMA was identified by patients, and mapped by the physician, at screening, baseline and end of study (EoS) in all patients. Mapping was performed using a brush to gently stroke the skin from outside the area indicated by the patient towards the centre, from six to eight directions (from above, below, left, right, etc.). A fixed velocity was not instructed for the brush strokes, as validated information for a suitable technique has not been published. Short strokes were used, based on clinical experience among the investigators, performed in a meticulous and careful manner with some seconds allowed for the patient to respond after each sweep. The exact location where the stroke became painful was marked on the skin. The points were joined with a dashed line to outline the perimeter of the area, which was traced onto a sheet of acetate. The acetate was then placed on graph paper and the squares (1 cm^2^) were counted to calculate the area.

To obtain the pain intensity score for DMA, the examiner applied three strokes to the centre of the allodynic area, maintaining the same direction and speed (about 5 cm per second) (Samuelsson et al., 2005) and purporting to maintain a constant force against the skin. A predetermined length of the stroke could not be provided, as patients with allodynic areas of varying sizes were entered into the study and, based on previous clinical observations of capsaicin 8% patch treatment, the possibility that the allodynic areas could shrink had to be considered. After the third stroke, the patient was asked to rate the intensity of the pain, evoked by the brush and independent of the ongoing background pain, using the 11‐point NPRS scale ranging from 0 (no pain) to 10 (worst imaginable pain).

### 2.3 Statistical methodology

The mean change per treatment group and difference between them were estimated using an analysis of covariance model adjusted for gender, baseline value (area/intensity of allodynia) and individual country (*post hoc* analysis). Least square (LS) means per treatment group and difference in LS means with associated 95% confidence interval (CI) and *p*‐value were provided. Baseline observation carried forward was applied in cases of missing data.

## 3 Results

### 3.1 Allodynia prior to treatment

Patient characteristics for DMA at screening and baseline were similar. At screening, patients randomized to treatment with the capsaicin 8% patch (*n *=* *240) had a mean (SD; standard deviation) intensity and area of DMA of 6.59 (1.82) and 223.0 (247.0) cm^2^, respectively. The corresponding figures for patients treated with pregabalin (*n *=* *225) were 6.70 (1.63) and 237.5 (283.1) cm^2^, respectively. At baseline, patients randomized to the capsaicin 8% patch (*n* = 253) had a mean (SD) intensity and area of DMA of 6.60 (1.66) and 228.3 (252.8) cm^2^, while the corresponding figures for pregabalin (*n* = 235) were 6.71 (1.58) and 234.0 (272.5), respectively. No significant differences were observed between the two treatment groups either at screening or baseline, or between screening and baseline in the intensity or area of DMA. The number of patients with mild allodynia (NPRS score <4) was the same in each treatment group (*n *=* *8).

### 3.2 Change in intensity of allodynia

**Figure 1:**
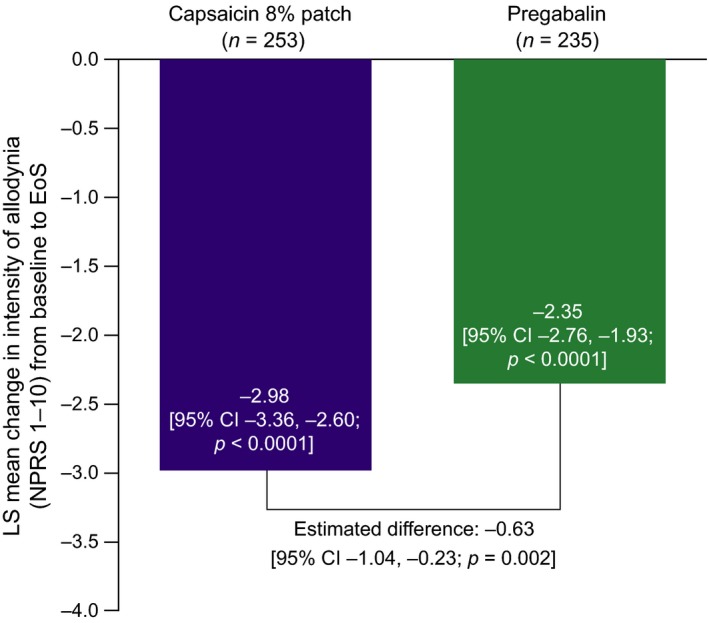
Mean change in intensity of dynamic mechanical allodynia from baseline to Week 8/EoS (BOCF). BOCF, baseline observation carried forward; EoS, end of study; LS, least squares; *n*, number of all patients with allodynia at baseline; NPRS, Numeric Pain Rating Scale.

The LS mean [standard error (SE)] change in intensity of DMA, from baseline to Week 8/EoS, was −2.98 (0.20) in the capsaicin 8% patch group and −2.35 (0.21) in the pregabalin group, significantly in favour of the capsaicin 8% patch [difference in LS means: −0.63 (95% CI: −1.04, −0.23; *p = *0.002)] (Fig. 1).

### 3.3 Change in area of allodynia

**Figure 2:**
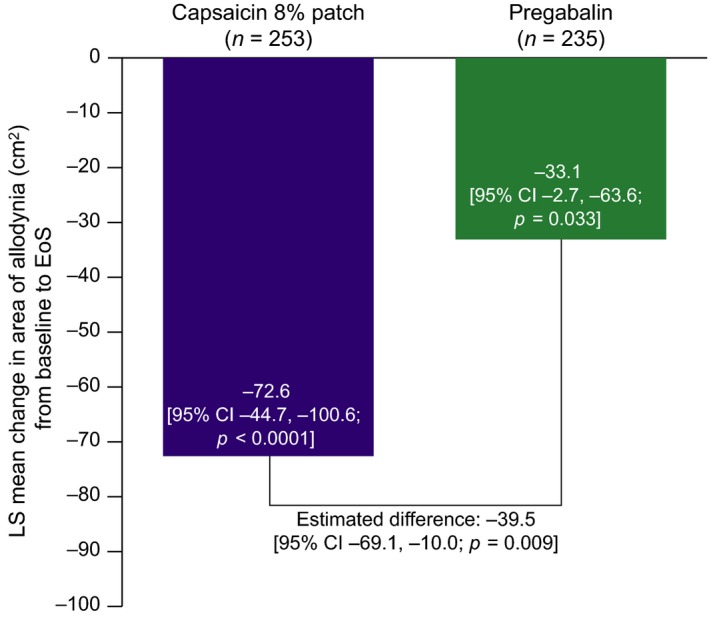
Mean change in area of dynamic mechanical allodynia from baseline to Week 8/EoS (BOCF). BOCF, baseline observation carried forward; EoS, end of study; LS, least squares; *n*, number of all patients with allodynia at baseline.

The LS mean (SE) change in area of DMA, from baseline to Week 8/EoS, was −72.6 cm^2^ (14.2) in the capsaicin 8% patch group and −33.1 cm^2^ (15.5) in the pregabalin group [difference in LS means: −39.5 cm^2^ (95% CI: −69.1, −10.0; *p =* 0.009)] (Fig. 2).

Significantly more patients had complete resolution of allodynia in the capsaicin 8% patch group compared with patients in the pregabalin group at Week 8/EoS (24.1% vs. 12.3%; *p = *0.001) (Table 1).

**Table 1:** Proportion of patients in each treatment group who experienced complete disappearance of dynamic mechanical allodynia at Week 8 (end of study)

Complete disappearance	Capsaicin 8% patch (*n = *253)	Pregabalin (*n = *235)	Significance^b^
Yes^a^, *n* (%)	61 (24.1)	29 (12.3)	*p = *0.001
No, *n* (%)	192 (75.9)	206 (87.7)	

*n*, number of all patients with allodynia at baseline.

a Defined as postbrush Numeric Pain Rating Scale = 0.

b The association between treatment and complete disappearance (chi‐square test).

### 3.4 Tolerability

In the ELEVATE study, fewer systemic side effects were observed with the capsaicin 8% patch compared with pregabalin (Haanpää et al., 2016). The majority of the treatment‐emergent adverse events with the capsaicin 8% patch were mild or moderate, and the most frequent were application site pain, application erythema and a burning at the site of application. Treatment‐emergent adverse events leading to permanent treatment discontinuation were only reported for pregabalin (*n = *24; 8.5%).

## 4 Discussion

Our study confirms that DMA, defined as pain evoked by brushing, is common in patients with peripheral NP, as previously reported (Attal et al., 2008; Baron, 2009; Maier et al., 2010; Jensen and Finnerup, 2014). In this head‐to‐head comparison study, significantly greater reductions in the intensity and area of DMA were seen after a single application of capsaicin 8% patch compared with optimized dose pregabalin over 8 weeks. Furthermore, the superiority in efficacy was not restricted to patients with mild allodynia (NPRS score <4), as these patients represented a minority (16 of 488) of the study population compared with those who had moderate‐to‐severe allodynia (NPRS score ≥4). Furthermore, capsaicin 8% patch was superior to pregabalin in the proportion of patients whose allodynia was completely controlled at EoS (Table 1). This improvement is of clinical importance when considering the functional disability that can be caused by allodynia and the lack of recognized treatment options (Svendsen et al., 2005; Jensen and Finnerup, 2014).

Topical capsaicin acts directly on TRPV1 membrane receptors of nociceptors (C and A‐delta) to induce defunctionalization of the peripheral terminals (Anand and Bley, 2011). C‐nociceptor input into the CNS is considered a key mechanism whereby central sensitization of second‐order neurons is maintained (Baron et al., 2013). With reduced C‐nociceptor input, centrally maintained activation of nociceptive pathways is lessened or removed. However, it is important to note that although the density of epidermal nerve fibres (predominantly C fibres) is usually reduced in painful peripheral NP, it is higher in those with DMA compared with those without (Truini et al., 2014). DMA may be maintained by excessive activity in spared C‐nociceptors as suggested by a study using laser‐evoked potentials (Truini et al., 2013a).

It has also been proposed that DMA could be mediated *directly* by activation of normally dormant low‐threshold C mechanoreceptors or by irritable nociceptors, that is high‐threshold mechanothermal C‐nociceptors with thresholds lowered by peripheral sensitization to the point of responding to low‐intensity stimuli, such as a gentle brushing. In agreement with this, microneurographic studies showed that light mechanical stimulation abnormally activates C‐nociceptors in painful polyneuropathy (Kleggetveit et al., 2012).

Although TRPV1 receptors are expressed in C‐ and A‐delta afferents in naïve animals only, it has been suggested that non‐nociceptive myelinated fibres can be expressed following nerve injury (Zakir et al., 2012). However, it remains to be shown if this happens in A‐beta fibres in human subjects and if this leads to DMA. Indirect evidence in support of this possibility comes from a study involving a small number of patients (*n* = 20) with peripheral NP treated with capsaicin 8% patch (Mainka et al., 2016). While a significant elevation of warm detection threshold was detected in the affected skin area following capsaicin treatment, it did not correlate with reported pain relief (Mainka et al., 2016). A further potential mechanism for C afferent dependent mechanical allodynia comes from evidence of C‐ and A‐beta fibre cross‐excitation in the dorsal root ganglia in neuropathic animals (Amir and Devor, 2000). Putatively, defunctionalization by topical capsaicin could attenuate the hyperexcitable C afferents in this mechanism. However, no studies have been published to date to indicate whether this mechanism is present in patients experiencing pain.

While the ELEVATE study demonstrated noninferiority in overall pain reduction between pregabalin and the capsaicin 8% patch, the latter was clearly superior in relieving DMA and was associated with fewer treatment‐emergent adverse events (Haanpää et al., 2016). Pregabalin is known to be efficacious in allodynia/hyperalgesia by modulating the calcium inflow into presynaptic terminals of primary afferents and thus reducing central sensitization (Tuchman et al., 2010), that is only *indirectly*. We propose that capsaicin was specifically superior in relieving allodynia because it can act both *indirectly* (by reducing central sensitization) and *directly* (by reducing peripheral sensitization). These are the first data to indicate a direct role for capsaicin in relieving DMA.

We acknowledge limitations of the ELEVATE study including challenging recruitment, open‐label design and limited study duration (Haanpää et al., 2016). More specific limitations concern the methods for evoking and measuring DMA over three separate sessions. To maximize accuracy and repeatability while accommodating the demands of a multicentre study, we adopted the common method for evoking tactile allodynia using a hand‐held light‐weight brush (Samuelsson et al., 2011). Previous studies show that in patients with peripheral neuropathy, the intensity of manually evoked brush allodynia is highly repeatable in several measurements made over a 30‐day period, with intraclass correlation coefficients ranging from 0.89 to 0.95, when the direction, velocity and the length of the brush sweep and the number of strokes and force exerted by the brush on the skin are kept constant (Samuelsson et al., 2005, 2007, 2011). This fact was emphasized to the examiners who also received detailed instructions for applying the appropriate test technique at investigator meetings. Patients were reminded to rate the brush‐evoked pain independently of the background pain. The method for assessing the area of allodynia was based on the patient's ability to reliably judge the margin at which nonpainful brushing becomes clearly painful. The identification of the boundaries of the allodynic areas has been successfully used in the past for both experimentally induced and clinical allodynia, although we are not aware of formal validation studies (Harding et al., 2001; Sjolund et al., 2001). In this study, allodynic areas measured at screening and baseline were remarkably similar. In addition, in a significant percentage of patients, the measurement at EoS indicated complete disappearance of DMA, making a powerful argument for a genuine impact of capsaicin 8% patch on DMA.

Caution should be used in extrapolating the results to other forms of allodynia in neuropathic and/or non‐neuropathic conditions. Future studies should address the issue of various subclasses of allodynia and hyperalgesia (Backonja et al., 2013) as well as the time to onset of relief from allodynic pain. Such studies should be based on a large number of known mechanisms for evoked pain (Meacham et al., 2017) and be adequately powered. The recent mechanistic study on the effects of capsaicin 8% patch that showed no impact on thermal and punctate hyperalgesia (or, indeed DMA) was likely underpowered (Mainka et al., 2016).

In conclusion, this study indicates that the capsaicin 8% patch provides stronger clinical benefits than pregabalin in treating DMA (reduction in area, reduction in intensity and number of patients reaching complete removal of allodynia). Whereas pregabalin can only act on central sensitization, the superiority of the capsaicin 8% patch in DMA may be due to capsaicin's ability to act on both central and peripheral sensitization.

## Author contributions

All authors contributed to the analysis and interpretation of the data, critically revised the publication, and approved the final version for submission.
